# *MCDataset*: a public reference dataset of Monte Carlo simulated quantities for multilayered and voxelated tissues computed by massively parallel *PyXOpto* Python package

**DOI:** 10.1117/1.JBO.27.8.083012

**Published:** 2022-04-18

**Authors:** Miran Bürmen, Franjo Pernuš, Peter Naglič

**Affiliations:** a University of Ljubljana, Faculty of Electrical Engineering, Ljubljana, Slovenia; b Sensum d.o.o., Ljubljana, Slovenia

**Keywords:** light propagation modeling, Monte Carlo, open-source, dataset, sampling volume, light scattering, phase function, light-tissue interaction

## Abstract

**Significance:**

Current open-source Monte Carlo (MC) method implementations for light propagation modeling are many times tedious to build and require third-party licensed software that can often discourage prospective researchers in the biomedical optics community from fully utilizing the light propagation tools. Furthermore, the same drawback also limits rigorous cross-validation of physical quantities estimated by various MC codes.

**Aim:**

Proposal of an open-source tool for light propagation modeling and an easily accessible dataset to encourage fruitful communications amongst researchers and pave the way to a more consistent comparison between the available implementations of the MC method.

**Approach:**

The *PyXOpto* implementation of the MC method for multilayered and voxelated tissues based on the Python programming language and PyOpenCL extension enables massively parallel computation on numerous OpenCL-enabled devices. The proposed implementation is used to compute a large dataset of reflectance, transmittance, energy deposition, and sampling volume for various source, detector, and tissue configurations.

**Results:**

The proposed *PyXOpto* agrees well with the original MC implementation. However, further validation reveals a noticeable bias introduced by the random number generator used in the original MC implementation.

**Conclusions:**

Establishing a common dataset is highly important for the validation of existing and development of MC codes for light propagation in turbid media.

## 1 Introduction

Light propagation modeling in turbid media such as biological tissues is essential in many branches of biomedical optics, allowing estimation of various light-related physical properties that can be measured and further related to the composition and structure of biological tissues. Through the years, light propagation modeling has found its use in diffuse optical tomography,^1^^–^^3^ optoacoustic imaging,^4^^,^^5^ spatial frequency domain imaging (SFDI),^6^^,^^7^ hyperspectral imaging,^8^ tissue and sample characterization with integrating spheres,^9^^,^^10^ light delivery and treatment planning,^11^^,^^12^ and many more.

While light propagation in biological tissues is mathematically well-described by the radiative transfer equation,^13^ its analytical or approximate solutions are usually constrained to idealized, and many times impractical, geometries. For example, in one of our studies, we have shown that the material layout comprising an optical probe tip at the probe–tissue interface, which cannot be straightforwardly described by an analytical model, significantly affects the acquired reflectance.^14^ Moreover, approximate solutions such as the diffusion approximation are known to be inaccurate near tissue boundaries and light sources, and tissues with high absorption coefficients.^9^ To alleviate the limitations of the analytical and approximate solutions, the radiative transfer equation can be solved numerically using the stochastic Monte Carlo (MC) method.^15^^,^^16^ While at the very start the MC methods were burdened by a significant computational cost, requiring hours or days for the simulations to complete, massive parallelization achieved by graphics processing units (GPUs) enabled the MC method to become a reference standard in the field of biomedical optics.^17^^,^^18^ In the last decade, the MC method has seen an immense advancement in various aspects. Apart from the massive parallelization achieved by GPUs, the MC method has been advanced to model light propagation in heterogeneous configurations of biological tissues using voxel-based^18^^–^^20^ and mesh-based^21^^–^^25^ approaches and has become more time-efficient for solving inverse problems for estimation of optical properties of turbid media attributed to the rapid development in hardware and deep learning approaches.^26^^,^^27^

In recent years, numerous free and open-source light propagation modeling packages based on the stochastic MC method have emerged. Most notably, the Fang group has a track record of successful publications that established and developed the well-known voxel- and mesh-based MC methods for accurate simulations of light propagation in complex tissue structures.^18^^,^^21^^,^^28^ FullMonte is another excellent mesh-based MC method package developed by the Lilge and Betz group,^24^ who have recently also introduced a parallelized version based on CUDA.^29^ Recently, Leino et al.^25^ have proposed a mesh-based MC software ValoMC interfaced with MATLAB^®^ for easier utilization. In the interest of user-friendliness and use in an educational environment, Marti et al.^30^ have developed an MCmatlab light transport solver with heat-diffusion and tissue-damage simulator. While all the proposed implementations have undoubtedly contributed to wider acceptance and utilization of the MC method, the implementations either involve complex installation steps or depend on licensed software such as MATLAB. Consequently, the adaptation of the free and open-source MC-based light propagation packages is still limited in the biomedical optics community.

On the other hand, the Python programming language is becoming more and more popular in the scientific community, offering a seamless interface to deep learning, visualization, and highly optimized numerical tools for scientific computations. Therefore, Python is an ideal candidate for establishing a collection of computational tools offering cross-platform, freely available, and user-friendly software that can be shared and distributed through various platforms. Moreover, Python-based tools can be used in a straightforward manner to introduce students and prospective researchers to light propagation modeling.

In addition to the limited availability of the open-source and user-friendly MC implementations, there is also a lack of a good reference dataset comprising estimated physical quantities that can be used for the validation of custom-developed MC methods. At the moment, such validation usually includes tedious investigation of the available open-source implementations, which often require time-consuming preparation of specific input files and processing of data. Moreover, such a dataset can serve as a quick-access lookup table for various estimated physical quantities without requiring MC simulations being run on a local computing machine.

In this paper, we briefly introduce a user-friendly open-source Python package *PyXOpto* offering light propagation in multilayered or voxelated turbid media based on the stochastic MC method.^31^ Subsequently, we propose and describe an open dataset *MCDataset*, which comprises simulated physical quantities such as total and spatially resolved reflectance and transmittance, energy deposition, sampling volume (SV), and reflectance in the spatial frequency domain. Finally, the data from the *MCDataset* is validated using the original MC code^16^ for multilayered tissues (MCML) and some examples of the simulated physical quantities from the *MCDataset* are presented.

## 2 Software Package *PyXOpto* and Datasets

### 2.1 Description of the Open-Source Package *PyXOpto*

#### 2.1.1 General structure of the open-source package *PyXOpto*

**Fig. 1:**
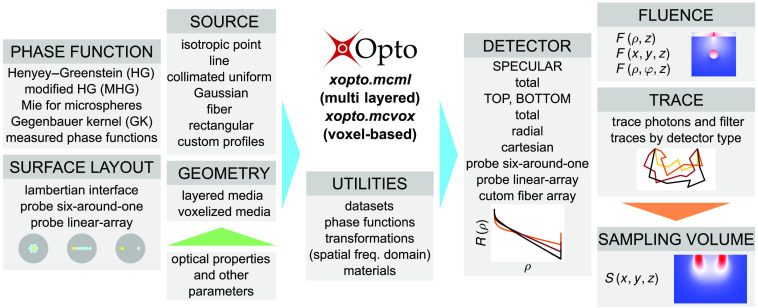
Schematic of the modular structure of the open-source package *PyXOpto*. Boxes represent available modules within the subpackages *xopto.mcml* and *xopto.mcvox* or helper utilities.

The general outline of the package *PyXOpto* is shown in Fig. 1. While the light propagation core is based on the PyOpenCL library and written in OpenCL C extension, the supporting modules are entirely Python-based and form a highly modular structure of the package *PyXOpto*. The PyOpenCL library enables massively parallel computations on OpenCL-enabled devices such as AMD, Intel, Nvidia, Qualcomm, ARM, and more. The light propagation core offers customizable kernel types that can perform single- or double-precision computations and use 32- or 64-bit photon packets counters. This allows more than the usual $2^{32}$ ($4.29\times 10^{9}$) photon packets to be processed in a single run.

The package *PyXOpto* is divided into two subpackages for modeling the light propagation in multilayered (*xopto.mcml*) and voxel-based (*xopto.mcvox*) configurations of tissues. Both *xopto.mcml* and *xopto.mcvox* share the same principle by using a central module *mc* from which the constructor *Mc* can be passed several modules that define the geometry, phase functions, surface layout of the top or bottom tissue boundary, sources, detectors, fluence accumulators, and trace accumulators. This configuration makes the *PyXOpto* package highly flexible since various combinations of modules can be used interchangeably and seamlessly without modifying the central light propagation core. In addition, the package *PyXOpto* offers various utilities for easier calculations of SVs, a transformation of reflectance to a spatial-frequency domain, scattering phase functions, and calculations of their subdiffusive quantifiers^32^^–^^34^ (e.g., Legendre moments, γ, δ, and σ) and various materials (e.g., wavelength-dependent refractive indices). The utilities also include a module for computing and accessing the presented *MCDataset* or generating new datasets for custom source, detector, and tissue configurations.

Both *xopto.mcml* and *xopto.mcvox* subpackages follow the standard propagation logic of the photon packets as used in the MCML.^16^ The details regarding each of the previously mentioned modules to the *Mc* constructor are described in the sections below.

#### 2.1.2 Geometry

The geometry description depends on the selection of the *PyXOpto* subpackages.

For the *xopto.mcml*, layers are defined in a similar fashion to the MCML except for the phase function that is further detailed in Sec. 2.1.3. For each layer, the user defines optical properties including the absorption and scattering coefficients, refractive index, the thickness of the layer, and phase function. The topmost and bottommost layers correspond to the media surrounding the tissue and supply only the refractive index mismatch, while other layers are used for photon packet propagation.

For the *xopto.mcvox*, the three-dimensional (3D) volume is labeled using a 3D matrix and for each label, the optical properties are defined. Generally, each voxel can be assigned a different absorption and scattering coefficient, refractive index, and phase function.

#### 2.1.3 Phase function

One of the stronger suits of the proposed light propagation model is that we do not strictly enforce the generic Henyey–Greenstein (HG) phase function.^35^ While most of the currently available free and open-source implementations of the MC method indeed offer only sampling of the scattering angles according to the HG phase function, such a restriction can significantly limit the applicability of the light propagation model for tissues exhibiting microscopic refractive index variations that cannot be accounted for by a single-parameter HG phase function. We have noted in several of our previous publications that the phase functions significantly influence the acquired reflectance at small source–detector separations when using optical fiber probes.^33^^,^^36^ Therefore, we have implemented a general lookup-table-based sampling scheme for phase function with cumulative probability distributions that have no analytical inverse.^37^ Such an approach supports the utilization of various scattering phase functions, such as the modified versions of the HG,^32^ Gegenbauer kernel,^38^ and Power of Cosines,^32^ measured phase functions, and more complex scattering phase functions based on the Mie theory.^39^ The latter can be used to simulate light propagation through water-based suspension of spherical microparticles, such as polystyrene microspheres and is therefore ideally suited for the fabrication of optical phantoms.

The phase function is used as an input to the geometry module that describes the biological tissue in which the light propagation is modeled. In general, for each layer or voxel, a different phase function can be applied thus enabling various complex configurations of tissues with spatially varying microstructure.

#### 2.1.4 Surface layout

The surface layout is an optional module and offers a realistic description of the interface between the tissue and the surrounding medium such as a Lambertian surface or a realistic layout of the optical fibers and materials comprising the tip of common commercially available optical fiber probes. We have found that especially the optical probe tip can significantly affect the acquired reflectance and thus the probe-tissue interface must be precisely described.^14^ To the best of our knowledge, the existing MC method implementations lack support for this important feature. If the surface layout is not set, the simulations are run with laterally uniform boundaries, which is the “standard” way of performing MC simulations in multilayered tissues.

#### 2.1.5 Source

The source is a mandatory input to the *Mc* constructor. We have implemented an isotropic source, line source, collimated Gaussian and uniform beams, optical fiber source, and rectangular sources that can be patched together to form more complex sources. All the sources require their specific parameters. For example, the optical fiber source depends on the diameter, position, direction, refractive index, and numerical aperture. Furthermore, the optical fiber and rectangular source can both accept a custom angular emittance profile measured with a goniometric setup. More realistic modeling conditions, especially custom emittance profiles, are useful for, e.g., health monitoring devices.

#### 2.1.6 Detector and fluence

Detection of photon packets can be performed using a surface detector or a 3D accumulator which we refer to as fluence.

Implemented surface detector can be divided into groups of specular, top, and bottom. Specular detectors merely record the fractions of the photon packets that have reflected due to the surrounding medium-tissue or source-tissue refractive index mismatch. Top and bottom sample surface allow different configurations of the detectors which can be selected from total detectors for quantities such as reflectance and transmittance, radial detectors for spatially resolved reflectance and transmittance, Cartesian detectors, detector configurations related to commercially available optical fiber probes such as the six-around-one and linear-arrays and finally a detector based on custom arrays of optical fibers for special use cases. As in the case of the source definitions, detectors accept various parameters including minimum acceptance cosines in the case of radial and Cartesian detectors and numerical aperture for fiber-based detectors. Finally, detectors can be specified with a custom angular acceptance measured with a goniometric setup.

The absorbed weight of the photon packets can also be recorded volumetrically using the so-called fluence accumulator. The latter can record energy deposition or true fluence depending on the additional flag that is specified to the constructor.

#### 2.1.7 Trace and sampling volume

The photon packets can optionally be traced by recording the photon packet position, direction, and weight at each absorption, scattering, or refraction event. The traces can be recorded independently of the selected detector and easily filtered according to the desired final direction and position of the photon packets. The filtered traces can be transformed into a voxelated SV, where each voxel contains path lengths of the photon packets multiplied by their respective terminal weights. The SV can be used to represent a 3D heat map indicating the probability of a photon packet to travel through a particular voxel from the source to the detector.^40^ The processing of the photon packet traces in our implementation utilizes the PyOpenCL implementation that significantly accelerates the computations of the SVs.

### 2.2 Structure and Parameter Specifications of the Datasets

In this section, we present the structure and parameters of the *MCDataset*. The *MCDataset* is segmented into five subsets, each corresponding to a specific configuration described in the following sections.

#### 2.2.1 *MCML comparison* dataset

**Fig. 2:**
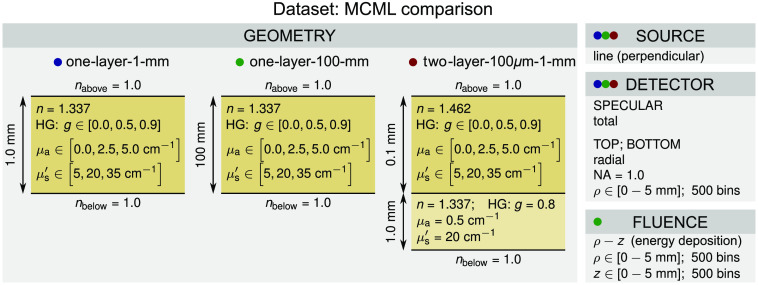
Configuration of the *MCML comparison* dataset. The colored dots correspond to different layer arrangements, thicknesses, and optical properties (see geometry).

The *MCML comparison* dataset is used for comparison to the MCML by Wang et al.^16^ It comprises three subsets, namely *one-layer-1-mm*, *one-layer-100-mm*, and *two-layer-100-*μm-*1-mm* corresponding to different layer arrangements and thicknesses, and optical properties (see Fig. 2, geometry). The source is an infinitely narrow perpendicular line (pencil beam). The optical properties of the single or top layer are varied across all combinations given by the outer product of the anisotropy factor g, absorption coefficient $\mu_{a}$, and reduced scattering coefficient $\mu_{s}^{\prime }$ (other optical properties and parameters remain constant). The specular detector stores total specular reflectance relative to the initial number of photon packets N. The radially accumulated photon packets at the top and bottom surface of the sample hold the spatially resolved reflectance and transmittance provided relative to N and per surface area of the annular ring (${1/m}^{2}$). Note that the transmittance is different from zero only for the *one-layer-1-mm* and *two-layer-100-*μm*-1-mm* subsets. The photon packets are collected for all exit angles (numerical aperture equal to 1.0). Finally, the energy deposition is stored in the fluence accumulator and is given relative to N and the volume of the voxel (${1/m}^{3}$). The *MCML comparison* dataset comprises 81 different configurations. Each configuration was simulated with $10^{8}$ photon packets.

#### 2.2.2 *MCML* dataset

**Fig. 3:**
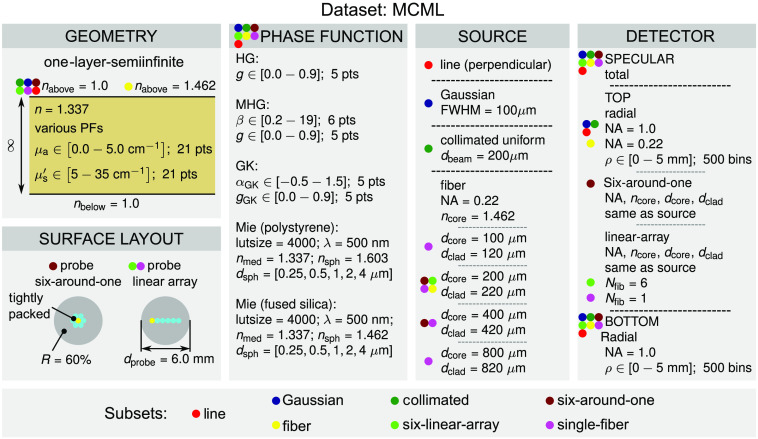
Configuration of the *MCML* dataset. The colored dots represent subsets of the MCML dataset with specifically assigned geometry, surface layout, source, and detector types.

The *MCML* dataset is considerably more extensive than the *MCML comparison* dataset and includes various phase functions, surface layouts, sources, and detectors. The dataset comprises seven groups of subsets that are color-coded and presented in Fig. 3. Each color represents a different source type. The number of subsets in each group depends on the number of different parameters defined for each source type. For example, the group corresponding to the collimated Gaussian beam source includes a single subset with the FWHM of the beam equal to 100  μm, while the group corresponding to the single-fiber source includes four subsets where the diameter of the fiber core and cladding varies from 100 and 120  μm to 800 and 820  μm. In total, the *MCML* dataset comprises 11 subsets. The subsets were selected according to various source and detector configurations that we have found in the literature.^41^^–^^43^ Each subset is assigned its corresponding geometry, surface layout, source, and detector according to the colored dots given in Fig. 3.

For example, the *fiber* dataset is based on a single semi-infinite layer geometry with the refractive index $n_{\text{above}}$ above the tissue equal to 1.462 representing a laterally uniform fiber-tissue boundary (no surface layout). Furthermore, the *fiber* dataset includes all the phase functions, a source with a fiber core and cladding diameter of 200 and 220  μm, the numerical aperture of 0.22 and refractive index of the core equal to 1.462, a detector of total specular reflectance, and top and bottom radial detectors with numerical apertures of 0.22 and 1.0, respectively, spanning from 0 to 5 mm with 500 bins. It should be noted that the refractive index $n_{\text{above}}$ is for the *fiber* subset deliberately set to 1.462 (fused silica). This way, the spatially resolved reflectance given by the radial detector can then be integrated to obtain an estimate of reflectance for a detector fiber at a given source–detector separation. In other cases, such as *six-around-one*, *six-linear-array*, or *single-fiber*, the proper probe-tissue boundary is achieved by the surface layout selection and thus the refractive index $n_{\text{above}}$ is set to 1.0 as it represents the refractive index of the air surrounding the metal housing of the probe. Photon packets incident within the area of the probe tip reflects from the metal housing with the reflectivity set to 60%. Finally, for the *six-linear-array* or *single-fiber*, the parameter $N_{\text{fib}}$ represents the number of fibers in the layout. For example, $N_{\text{fib}}=6$ corresponds to one fiber being used simultaneously as a source and detector and the remaining five fibers as detectors in a tightly packed manner (i.e., the smallest source–detector separation is equal to the diameter of the fiber cladding). As expected, the value of $N_{\text{fib}}$ for the single-fiber setup is set to 1.

The explanation for the parameters of various phase functions used in the dataset can be found in one of our recent publications.^37^ The Mie phase function was derived for polystyrene and fused silica microspheres ranging in diameter from 0.25 to 4  μm as given in Fig. 3. The refractive index of polystyrene and fused silica was obtained from Nikolov and Ivanov^44^ and Malitson^45^ for the wavelength of light equal to 500 nm. The surrounding medium was set to water with a refractive index of 1.337 obtained from Daimon and Masumura.^46^ All the refractive indices can be retrieved from the utility *materials* in the package *PyXOpto*.

The *MCML* dataset comprises 11 subsets corresponding to different sources, each simulated for 30,870 combinations of optical properties and phase functions, resulting in a total number of 339,570 configurations. Each configuration was simulated with $10^{8}$ photon packets except for the *linear-array* subset configuration, where $10^{9}$ photon packets were used to achieve an acceptable signal-to-noise ratio (SNR) at the most distant optical fiber.

#### 2.2.3 *MCVOX* dataset

**Fig. 4:**
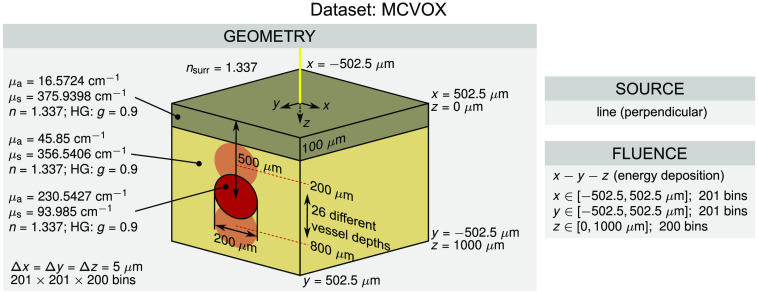
Configuration of the *MCVOX* dataset. Energy deposition is normalized by the product of the voxel volume and the total weight of the launched packets ($m^{-3}$).

The *MCVOX* dataset is based on a two-layered skin model with an embedded blood vessel. Light propagation modeling for this dataset is performed using the *xopto.mcvox* subpackage from *PyXOpto*. The optical properties of the epidermis, dermis, and blood vessel were obtained from Jacques^47^ and are given in Fig. 4 along with dimensional parameters of the skin model. In this dataset, a line (pencil beam) was used as a source. The energy deposition was accumulated using the fluence module and the reflectance and transmittance using a Cartesian type detector. The details of the voxelization of the energy deposition are given in Fig. 4. The *MCVOX* dataset comprises 26 geometrical configurations in which the depth of the embedded vessel is varied uniformly from 0.2 to 0.8 mm as is illustrated in Fig. 4. Each configuration was simulated using $10^{9}$ photon packets.

#### 2.2.4 *SV* dataset

**Fig. 5:**
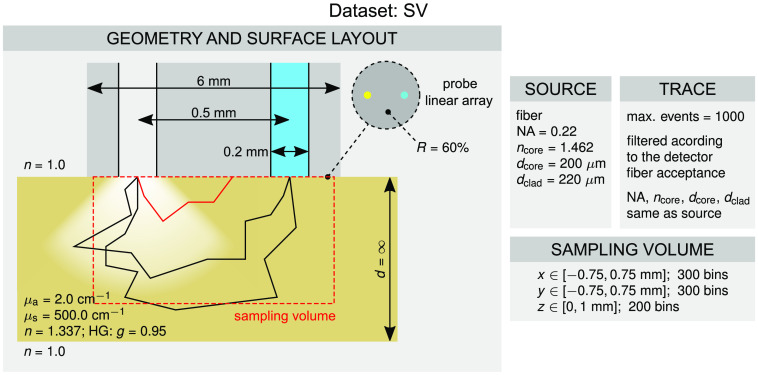
Configuration of the *SV* dataset.

The *SV* dataset is based on a single geometrical configuration given in Fig. 5 and comprises an SV for an optical fiber probe with the source and detector fibers separated by 500  μm. The two fibers are embedded in a metal housing with reflectivity of 60% that is implemented through the surface layout module. The dimensions of the probe and optical properties of the tissue are further given in Fig. 5. The photon packet traces were recorded by storing up to 1000 events per photon packet. It should be noted that the capacity of the trace can be changed and was in this case determined beforehand since the photon packets that reach the detector rarely undergo >1000 events. The traces of the photon packets were filtered using the specifications of the detector fiber, which were the same as for the source fiber, i.e., numerical aperture equal to 0.22, core refractive index equal to 1.462, and core and cladding diameters equal to 200 and 220  μm. The SV was then prepared using the embedded utility of the *PyXOpto* package. The spans and number of bins for the voxelization of the SV are given in Fig. 5.

#### 2.2.5 *SFDI* dataset

**Fig. 6:**
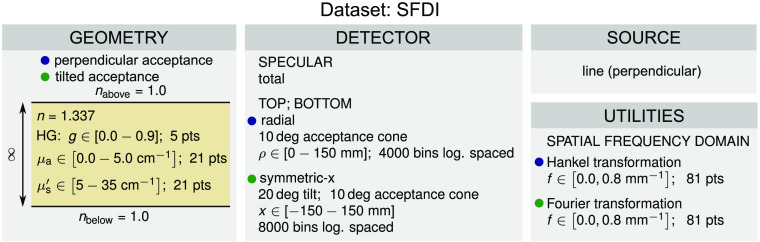
Configuration of the *SFDI* dataset.

The *SFDI* dataset comprises two subsets of simulated spatial frequency domain reflectance spanning spatial frequencies from 0 to $0.8\;{\mathrm{mm}}^{-1}$ using 81 uniformly distributed points. The two subsets are color-coded in Fig. 6 and differ in terms of the detectors. Detectors in the first subset include total specular reflectance and top and bottom spatially resolved reflectance acquired perpendicularly to the tissue with a 10 deg acceptance cone using 4000 logarithmically spaced radially symmetric bins spanning from 0 to 150 mm. We have recently shown that such a configuration is necessary for the accurate discrete transformation of the spatially resolved reflectance into the spatial frequency domain.^48^ While the detectors in the second subset also include the total specular reflectance, the spatially resolved reflectance is acquired through a detector tilted by 20 deg with an acceptance cone equal to 10 deg using 8000 logarithmically spaced x-symmetric bins (infinite in the y-direction) spanning from −150 to 150 mm. The latter configuration more accurately represents the realistic case where the tissue is illuminated perpendicularly, and the backscattered light is acquired with a tilted camera. Finally, it should be noted that for the first subset the spatial frequency domain reflectance can be obtained using the Hankel transformation since the spatially resolved reflectance is radially symmetric. For the second subset, one-dimensional Fourier transformation must be performed along the x-direction, since the spatially resolved reflectance is symmetric across the x-axis. The spatial frequency domain reflectance is unitless. The *SFDI* dataset comprises 2205 different configurations of optical properties for each subset resulting in a total number of 4410 configurations. Each configuration was simulated with $10^{9}$ photon packets to yield an acceptable SNR at long distances from the source.

#### 2.2.6 Organization of the *MCDataset*

The *MCDataset* is publicly available on GitHub (https://github.com/xopto/mcdataset) and can be generated, customized, and accessed using the dataset utility within the *PyXOpto* package.

## 3 Validation of the *PyXOpto* Package and Examples from the *MCDataset*

### 3.1 Validation of the *xopto.mcml*

The subpackage *xopto.mcml* was validated by comparing the total reflectance and transmittance, spatially resolved reflectance and transmittance, and energy deposition to the values computed by the MCML. For this purpose, we have utilized the *MCML comparison* dataset, which was also simulated with the MCML using the same configuration and $10^{7}$ photon packets. All the relative differences between the *xopto.mcml* and MCML were calculated by subtracting the results of the MCML from the *xopto.mcml* and dividing the difference by the results of *xopto.mcml*. This way, the relative error was less affected by the simulation noise that was present due to a smaller number of photon packets that were launched during the simulations with the MCML.

#### 3.1.1 Total reflectance and transmittance

**Fig. 7:**
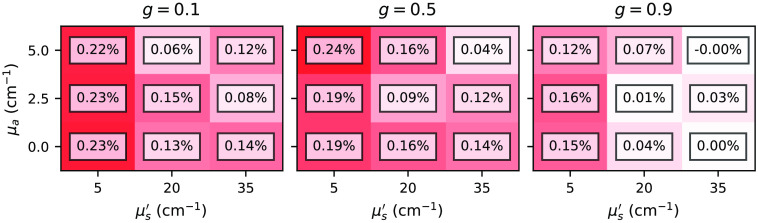
Relative errors between the *xopto.mcml* and MCML simulations of the total reflectance for the dataset *MCML comparison* and subset *two-layer-100-*μm*-1-mm*.

**Fig. 8:**
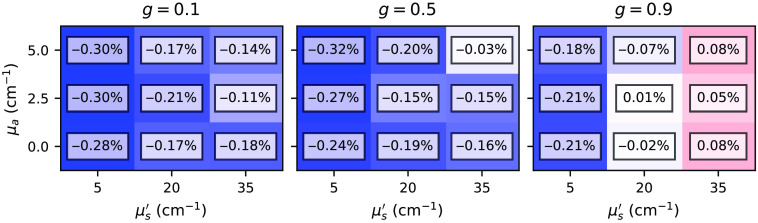
Relative errors between the *xopto.mcml* and MCML simulations of the total transmittance for the dataset *MCML comparison* and subset *two-layer-100-*μm*-1-mm*.

Figures 7 and 8 show relative differences between the total reflectance and transmittance for the subset *two-layer-100-*μm*-1-mm* with various combinations of $\mu_{a}$, $\mu_{s}^{\prime }$, and g varied in the top layer. In principle, we can observe excellent agreement between the estimations is given by the *xopto.mcml* and the MCML for the total reflectance and transmittance. The absolute relative errors never exceed 0.32%. However, we do observe a slight positive and negative bias in the total reflectance and transmittance, which we attribute to the choice of the random number generator (RNG) in the MCML code, which is further discussed in Sec. 3.1.2. Figures 17 and 18 in the Appendix show that replacing the RNG in the MCML code with a cryptographic-grade RNG reduces the relative differences to negligible values. Note that subsets *one-layer-1-mm* and *one-layer-100-mm* exhibited similar errors.

#### 3.1.2 Spatially resolved reflectance and transmittance

**Fig. 9:**
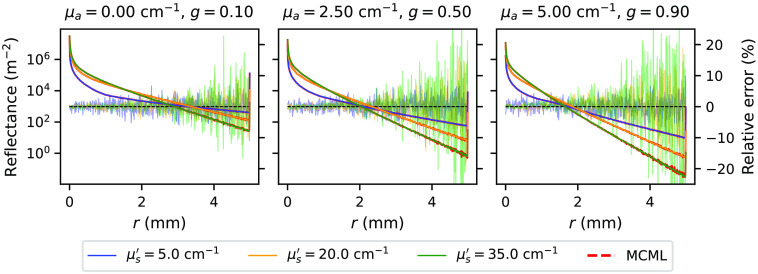
Spatially resolved reflectance as simulated by the *xopto.mcml* (black, blue, and green solid lines) and MCML (red dashed lines) for various combinations of optical properties $\mu_{a}$, $\mu_{s}^{\prime }$, and g in the dataset *MCML comparison* and subset *one-layer-1-mm*. Relative errors are denoted with the same colors. Scale is provided at the right-hand side of the graphs.

**Fig. 10:**
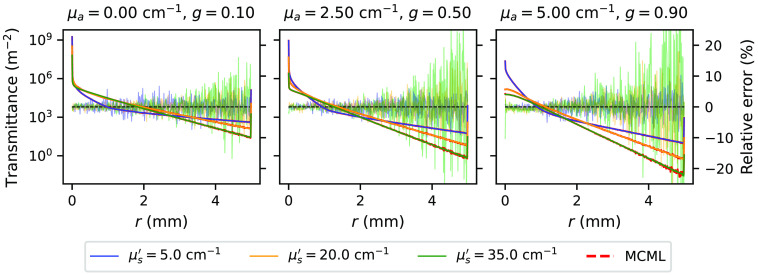
Spatially resolved transmittance as simulated by the *xopto.mcml* (black, blue, and green solid lines) and MCML (red dashed lines) for various combinations of optical properties $\mu_{a}$, $\mu_{s}^{\prime }$, and g in the dataset *MCML comparison* and subset *one-layer-1-mm*. Relative errors are denoted with the same colors. Scale is provided at the right-hand side of the graphs.

Figures 9 and 10 show spatially resolved reflectance and transmittance for the subset *one-layer-1-mm* for various combinations of optical properties $\mu_{a}$, $\mu_{s}^{\prime }$, and g. The spatially resolved reflectance and transmittance completely overlap with the calculated relative errors indicating no bias. The relative errors are higher at longer distances due to the simulation noise.

#### 3.1.3 Energy deposition and effect of the random number generator

**Fig. 11:**
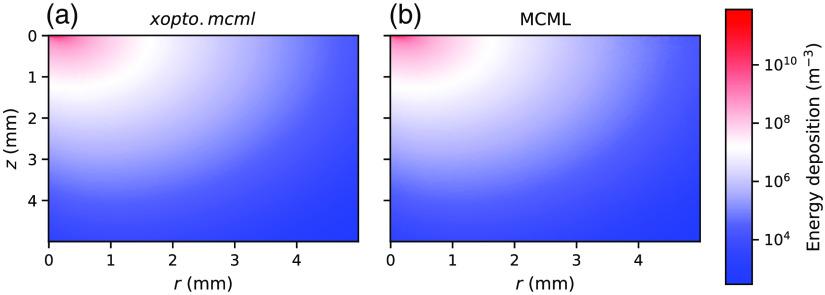
Deposited energy map simulated by (a) *xopto.mcml* and (b) MCML for $\mu_{a}=2.5\;{\mathrm{cm}}^{-1}$, $\mu_{s}^{\prime }=30\;{\mathrm{cm}}^{-1}$, and g=0.9.

**Fig. 12:**
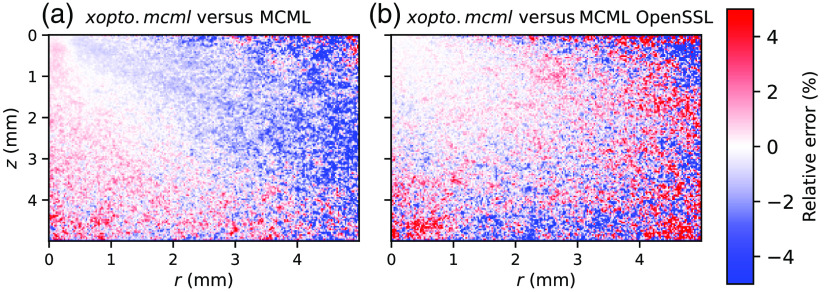
The relative errors between the energy deposition maps simulated by (a) *xopto.mcml* and MCML and (b) *xopto.mcml* and MCML with a modified RNG based on the cryptographic-grade RNG of the OpenSSL library. Deposited energy was simulated for a medium with $\mu_{a}=2.5\;{\mathrm{cm}}^{-1}$, $\mu_{s}^{\prime }=30\;{\mathrm{cm}}^{-1}$, and g=0.9.

**Fig. 13:**
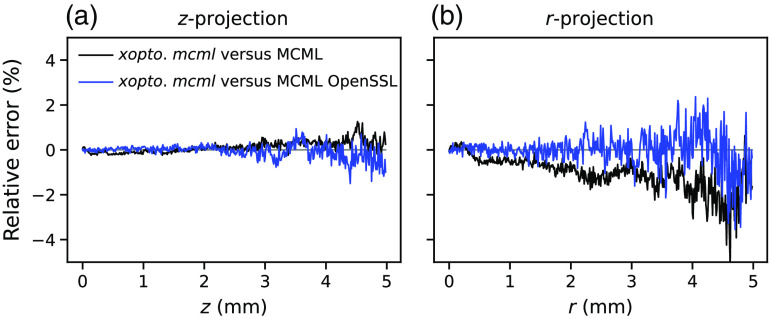
Relative errors between the energy deposition projected onto the (a) x and (b) r axis. The black solid line represents relative differences between *xopto.mcml* and MCML, while the blue solid line represents relative differences between the *xopto.mcml* and MCML with a modified RNG based on the cryptographic-grade RNG of the OpenSLL library.

The energy deposition maps as simulated by the *xopto.mcml* and MCML are given in Fig. 11. While the maps seem similar and show no apparent differences, Fig. 12(a) clearly shows a pronounced positive and negative bias. This bias is even more pronounced in Fig. 13 (black solid line), which shows the relative errors of the energy deposition maps projected onto the z or r axis.

Further investigation of the MCML code showed that the bias is apparently due to the RNG *ran3* from the Numerical Recipes in C.^49^ To confirm our hypothesis, we have modified the MCML code to accept large sets of random bytes that can be generated using for example cryptographic-grade RNG of the OpenSSL library.^50^ Using the random numbers generated by the latter RNG and repeating the simulations of the dataset *MCML comparison* removed all the apparent bias present in the total reflectance and transmittance (Figs. 7 and 8) and energy deposition in Fig. 11. This is confirmed by Figs. 12(b) and 13 (blue line) which show that the remaining errors are mostly governed by the simulation noise.

### 3.2 Validation of the *xopto.mcvox*

The subpackage *xopto.mcvox* was validated using the simulated total reflectance and transmittance and energy deposition as obtained by the subpackage *xopto.mcml* for the dataset *MCML* comparison. We, therefore, generated the same relative error maps as given in Figs. 7, 8, and 12 in which the estimations by the *xopto.mcvox* were subtracted from the *xopto.mcml* and the difference divided by *xopto.mcml*. Figures 19–22 given in the Appendix show no apparent bias in the errors between the *xopto.mcml* and *xopto.mcvox* simulated total reflectance, transmittance, and energy deposition thus proving that the *xopto.mcvox* essentially performs equally well as the previously validated subpackage *xopto.mcml*.

### 3.4 Energy Deposition from the MCVOX Dataset

**Fig. 14:**
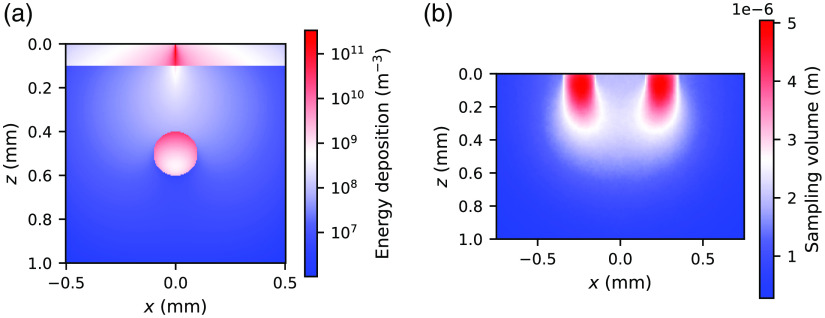
(a) Energy deposition averaged along the y-direction simulated for a two-layered skin model with an embedded vessel utilizing the *xopto.mcvox*. The whole MCVOX dataset is available as Video 1 (mp4, 130 kB) and shows the change in the averaged energy deposition when the depth of the embedded vessel is varied from 0.2 to 0.8 mm. (b) SV averaged along the y-direction simulated for an optical fiber probe with a source–detector separation of 500  μm utilizing the SV utility in the *PyXOpto* package (Video 1, mp4, 130 kB [URL: https://doi.org/10.1117/1.JBO.27.8.083012.1]).

Figure 14(a) shows an example of the simulated energy deposition averaged along the y-direction for a two-layered skin model with the blood vessel embedded at $z_{\text{vessel}}=0.5\;\mathrm{mm}$ utilizing the subpackage *xopto.mcvox*. Note that due to the higher absorption coefficient of the top layer and the blood vessel, the energy deposition is clearly much higher in the two regions than in the bottom layer. The whole dataset *MCVOX* is presented as Video 1 which shows an animation of the projected energy deposition by varying the depth of the embedded blood vessel from 0.2 to 0.8 mm [see Fig. 14(a) for further details].

### 3.5 Sampling Volume from the SV Dataset

Figure 14(b) shows the SV averaged along the y-direction for an optical fiber probe with a source–detector separation of 500  μm as detailed in the SV dataset. The SV clearly indicates that the photon packets that contribute to the detected reflectance are more likely to travel along the “banana-shaped” path from the source to the detector fiber.

## 4 Computation Time

We compared the computation times of *xopto.mcml* and *xopto.mcvox* to other established massively parallel implementations of the MC method. The evaluation was done on a Windows 11 system (Intel^®^ Core^™^ i7-8700K 3.70 GHz, 64 GB DDR4, Nvidia GeForce RTX 2800Ti). The performance of the multilayered *xopto.mcml* was compared with CUDAMCML developed by Alerstam et al.,^17^^,^^51^ while the voxelated *xopto.mcvox* was compared with MCX 1.8 (v2020, Furious Fermion) Rev. a6bc5a developed by Fang and Boas.^18^^,^^52^ It is important to note that for a fair comparison all the implementations should do the same workload, which includes processing the same number of photon packets and producing the same SNR of the simulated quantities.

Since *xopto.mcml* and CUDAMCML utilize the same photon packet stepping method based on the variant of the Albedo–Weight (AW) method,^53^ they exhibit the same SNR of the simulated quantities. Both implementations were evaluated on a single configuration from the *MCML comparison* dataset with optical properties $\mu_{a}=0.0\;{\mathrm{cm}}^{-1}$, $\mu_{s}^{\prime }=35.0\;{\mathrm{cm}}^{-1}$, and g=0.9, which yield the longest computation time within the dataset. The computation times were estimated by launching $10^{8}$ photon packets per simulation and averaging three consecutive simulation runs. Based on the achieved computation times, which are given in Table 1, slightly better performance of *xopto.mcml* is observed over CUDAMCML. It should be noted that for the purpose of the comparison, the CUDAMCML code was recompiled for the GPU Nvidia GeForce RTX 2800Ti with 7.5 compute capability.

**Table 1:** Average computation times for (a) multilayered and (b) voxelated MC methods evaluated on a single configuration from the *MCML comparison* and *MCVOX* datasets, respectively. See text for further details.

(a)		(b)		
*xopto.mcml*	CUDAMCML	*xopto.mcvox* (AW)	*xopto.mcvox* (MBL)	MCX
26.2 s	32.1 s	4.54 s	5.16 s	7.95 s

On the other hand, *xopto.mcvox* offers three different selectable variants of the photon packet stepping methods based on the AW, Albedo–Reject (AR) and Microscopic Beer–Lambert law (MBL) methods, while MCX utilizes a variant of the MBL method.^53^ Although all the methods are equivalent and converge towards equal results, they differ in the way they calculate and deposit the energy. It should be noted that in *xopto.mcvox* the voxelated grids of the sample and fluence/deposition accumulators are in general fully independent and that the packets are propagated according to the sample grid. Our implementation of the AW method deposits the absorbed weight at extinction events that are fully independent of the voxelated deposition grid. The AR method, which is many times referred to as the ballistic method, deposits the full photon packet weight only at an absorbing extinction event which depends on the albedo (ratio of the scattering and extinction coefficients) and is also fully independent of the voxelated deposition grid. At the absorbing extinction event, the photon packet is also terminated. The MBL method deposits the absorbed energy at scattering events and at each crossing of the sample voxel boundary.

Since the AR method is known to require an extremely high number of photon packets for an adequate SNR, we more thoroughly compared the AW and MBL methods in terms of the SNR and computation times utilizing the *xopto.mcvox* and MCX implementations. Note that for an easier distinction, we use *xopto.mcvox* (AW) and *xopto.mcvox* (MBL) with the selected photon packet stepping method provided in parentheses. When the voxel size is significantly smaller than the mean free path length, the AW method less frequently accesses the global memory of the GPU to accumulate the absorbed energy and is, therefore, faster than the MBL method. However, this results in a lower SNR of the simulated energy deposition than in the case of the MBL method. We have found that this is exactly the case for the *MCVOX* dataset, where the voxel size is extremely small 5×5×5  μm. For example, the mean free path of the photon packet in the dermis (with optical properties $\mu_{a}=45.9\;{\mathrm{cm}}^{-1}$ and $\mu_{s}=356.5\;{\mathrm{cm}}^{-1}$) is approximately $l=1/(\mu_{a}+\mu_{s})=25\;\mu m$. Consequently, the AW and MBL methods were compared on a single configuration from the *MCVOX* dataset with the blood vessel embedded at $z_{\text{vessel}}=0.5\;\mathrm{mm}$ and with a larger voxel size of 50×50×50  μm (21×21×20  voxels). With the latter adjustment, AW and MBL methods should produce energy depositions of comparable quality and thus enable a fair comparison. It should be noted that the above-described drawback of the AW method does not apply to reflectance or transmittance simulations.

**Fig. 15:**
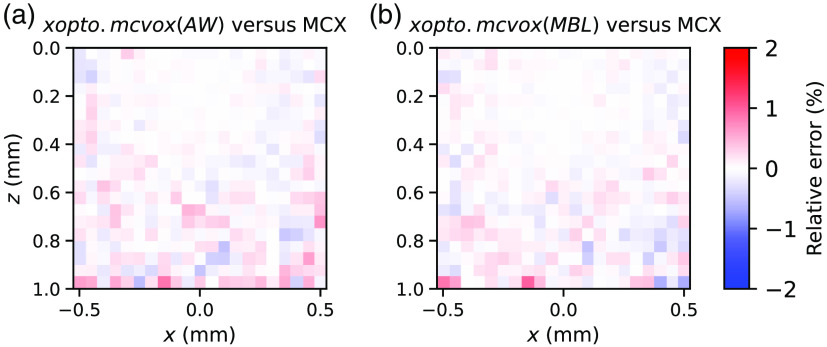
Relative errors of energy deposition at y=0  mm between (a) *xopto.mcvox* (AW) and MCX and (b) *xopto.mcvox* (MBL) and MCX.

**Fig. 16:**
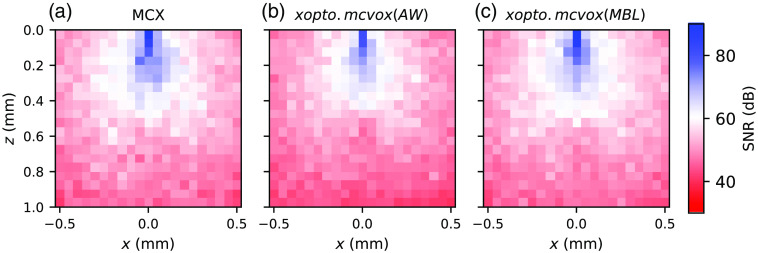
Relative SNR of energy deposition at y=0  mm as calculated by (a) MCX with a mean value of 53.76 dB, (b) *xopto.mcvox* (AW) with a mean value of 51.45 dB, and (c) *xopto.mcvox* (MBL) with a mean value of 53.62 dB. Relative SNR was calculated using 20 simulations with $10^{8}$ launched photon packets.

Figure 15 shows relative errors of energy deposition at y=0  mm for the *xopto.mcvox* (AR) [Fig. 15(a)] and *xopto.mcvox* (MBL) [Fig. 15(b)] in comparison to the MCX utilizing the MBL method. In both cases, a nice agreement is observed without any apparent bias between the two MC implementations and photon packet stepping methods. Furthermore, we evaluated the SNR distribution for *xopto.mcvox* and MCX, which was calculated as a ratio of the mean and standard deviation of 20 simulations of energy deposition at y=0  mm. Each simulation was performed by launching $10^{8}$ photon packets. The SNR distributions are shown in Fig. 16 and suggest that *xopto.mcvox* and MCX exhibit similar SNR. However, upon further evaluation of the mean SNR for each MC implementation and photon packet stepping method, we found that the *xopto.mcvox* (AW) exhibits a slightly lower SNR (51.45 dB) than *xopto.mcvox* (MBL) (53.62 dB) and MCX (53.76 dB).

Figures 15 and 16 suggest that the *xopto.mcvox* (MBL) and MCX yield energy deposition of similar quality, while the *xopto.mcvox* (AW) yields a slightly lower SNR of the energy deposition. With this in mind, we evaluated the computation times required to simulate energy deposition for a single configuration from the *MCVOX* dataset (as described above) by launching $2\times 10^{9}\text{ photon}$ packets and averaging three consecutive simulation runs. The number of threads and block size in the *xopto.mcvox* are chosen automatically by the OpenCL platform (usually termed as the workgroup size). The simulations by MCX were done by setting the flag “−A” to 1, which automatically determines the number of threads and block size. We have also observed that such a configuration is optimal for MCX. The benchmark input files that the readers can use to assess the computation times can be found here.^54^ According to the computation times provided in Table 1, slightly better performance of the *xopto.mcvox* is observed over the MCX regardless of the selected photon packet stepping method. However, it should be noted that a fair comparison can only be made between the *xopto.mcvox* (MBL) and MCX since *xopto.mcvox* (AW) exhibits a slightly lower SNR. To achieve the same SNR for the *xopto.mcvox* (AW), more photon packets would have to be launched, which would increase the computation time.

While we believe that the comparison of the computation times has been done objectively, the obtained values should be taken more as a demonstration that our proposed *PyXOpto* package can compete with the established and more modern multilayered and voxelated MC method implementations.

## 5 Conclusion

In this paper, we have presented a new open-source, user-friendly, and versatile tool *PyXOpto* that includes a light propagation model based on the MC method implemented for the multilayered and voxelated tissues and various utilities for processing and transforming the simulated physical quantities and providing various material properties such as refractive indices. Unlike the CUDA computing platform, which requires Nvidia GPUs, the light propagation core of the *PyXOpto* is based on the PyOpenCL package, which enables photon packet processing in a highly parallelized manner on various OpenCL-enabled devices. Finally, the modular structure of *PyXOpto* enables easy customization of the tissue geometry, phase functions, source, detectors, and other modules. To this end, *PyXOpto* offers simple simulations like the MCML and more complex voxelated tissue geometries with a realistic description of the source and detector configurations such as metal-housed optical fiber probes.

As a central part of the paper, we have proposed an openly available *MCDataset* that includes various configurations that can be easily accessed and used to validate custom-developed MC code. Since the proposed dataset is publicly available on the GitHub repository, it is possible for any member of the biomedical optics community or any other community for that matter to enter a discussion regarding a specific dataset or raise an issue when a certain dataset cannot be matched to the results of a custom-developed MC method. This is an important feature that should encourage fruitful communications between researchers inducing a pathway to a more consistent comparison between the available implementations of the MC method.

While the main benefit of the *MCDataset* should be easy access to validation data, *MCDataset* can in fact also be used for fast lookup of various physical quantities such as reflectance and transmittance from desired optical properties without having to run simulations. Since the optical properties in the *MCDataset* are densely distributed, physical quantities that do not correspond precisely to the ones given in the *MCDataset* can be approximated using interpolation.

Finally, it should be mentioned that the *PyXOpto* could be utilized as a teaching tool, allowing the easier introduction of MC methods to students and prospective researchers in biomedical optics.

## 6 Appendix

### 6.1 Comparison Between *xopto.mcml* and MCML with a Modified RNG

**Fig. 17:**
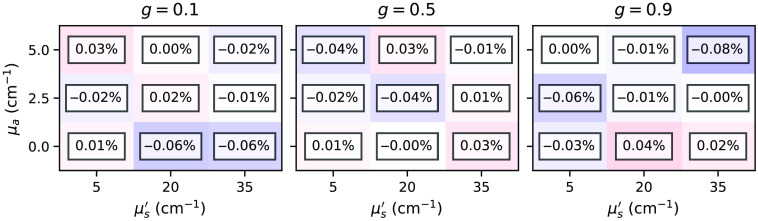
Relative errors between the total reflectance simulated by the *xopto.mcml* and MCML with a modified RNG for the dataset *MCML comparison* and subset *two-layer-100-*μm*-1-mm*.

**Fig. 18:**
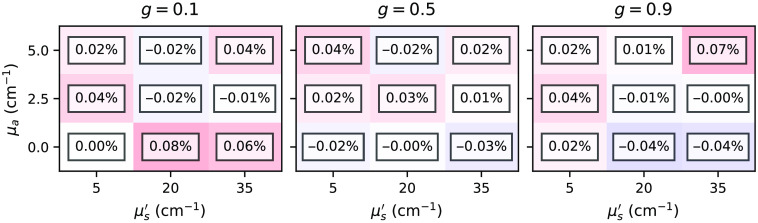
Relative errors between the total transmittance simulated by the *xopto.mcml* and MCML with a modified RNG for the dataset *MCML comparison* and subset *two-layer-100-*μm*-1-mm*

Figures 17 and 18 show relative errors between the total reflectance and total transmittance, respectively, from the dataset *MCML comparison* and subset *two-layer-100-*μm*-1-mm* as simulated by the *xopto.mcml* and MCML with a modified RNG based on the cryptographic grade RNG of the OpenSSL library. Note that the relative errors are virtually insignificant in comparison to Figs. 7 and 8.

### 6.2 Validation of the *xopto.mcvox*

**Fig. 19:**
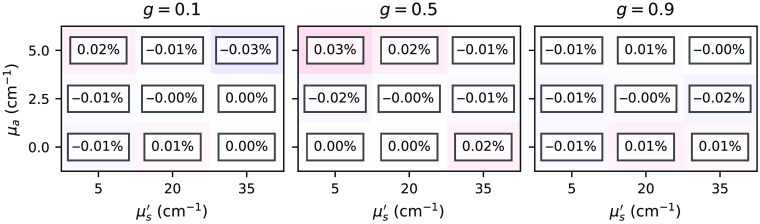
Relative errors between the total reflectance simulated by the *xopto.mcml* and *xopto.mcvox* for the dataset *MCML comparison* and subset *two-layer-100-*μm*-1-mm*.

**Fig. 20:**
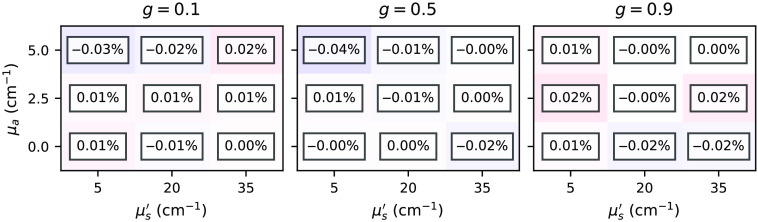
Relative errors between the total transmittance simulated by the *xopto.mcml* and *xopto.mcvox* for the dataset *MCML comparison* and subset *two-layer-100-*μm*-1-mm*.

**Fig. 21:**
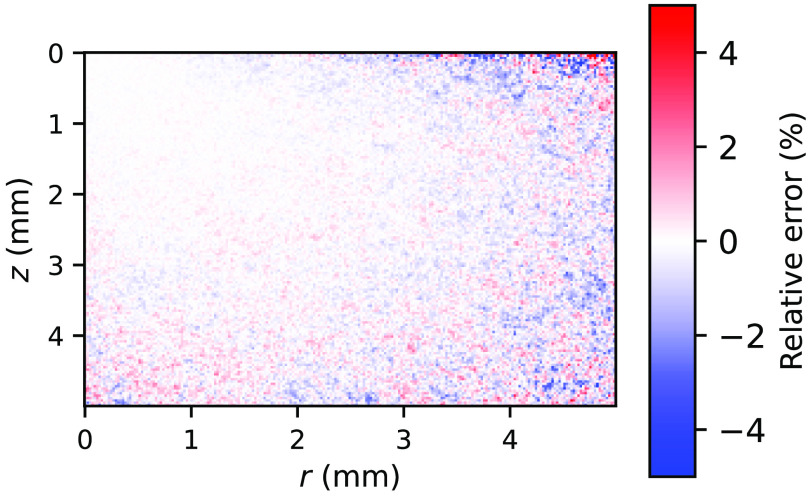
Relative errors between the energy deposition maps simulated by the *xopto.mcml* and *xopto.mcvox*.

**Fig. 22:**
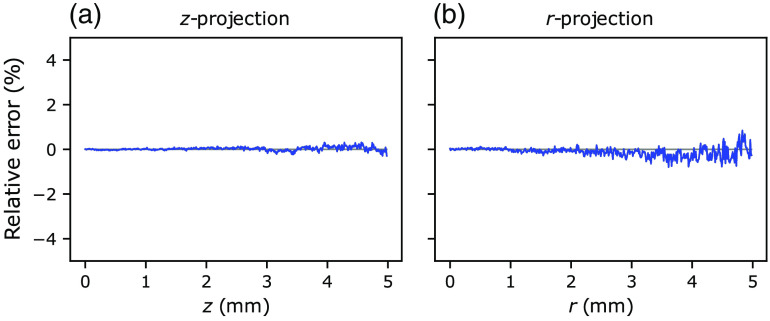
Relative errors between the energy deposition simulated by the *xopto.mcml* and *xopto.mcvox* projected onto the x (a) and r axis (b).

Figures 19–21 show relative errors between the total reflectance, total transmittance, and energy deposition, respectively, simulated by the *xopto.mcml* and *xopto.mcvox* for the dataset *MCML comparison* and subset *two-layer-100-*μm*-1-mm*. Note that the relative errors do not exhibit bias. The projected relative errors for the energy deposition are presented in Fig. 22.

## Code, Data, and Materials Availability

The package *PyXOpto* and *MCDataset* are available on Github (https://github.com/xopto/pyxopto and https://github.com/xopto/mcdataset).
